# Validation of the sleep disturbance scale for children (SDSC) in infants and toddlers from mainland China

**DOI:** 10.3389/fpsyt.2022.987304

**Published:** 2022-11-10

**Authors:** Xianrui Chen, Ping Xu, Yanhui Chen, Shan Chen, Yonghua Yao, Xiaoxia Lin

**Affiliations:** ^1^Department of Pediatric Rehabilitation, Xiamen Rehabilitation Hospital, Xiamen, China; ^2^Fujian Family Planning Association, Fuzhou, China; ^3^Department of Pediatrics, Fujian Medical University Union Hospital, Fuzhou, China; ^4^Pediatric Key Laboratory of Xiamen, Department of Pediatrics, The First Affiliated Hospital of Xiamen University, School of Medicine, Institute of Pediatrics, Xiamen University, Xiamen, China

**Keywords:** sleep, Chinese version, sleep disturbance scale for children, infants, toddlers

## Abstract

The sleep disturbance scale for children (SDSC) has been validated in the population of Chinese children (aged 5–16 years) and has good psychometric properties. However, valid assessment tools for sleep problems in the population of infants and young children from mainland China are relatively scarce, and rates of screening and management for pediatric sleep problems are low.

**Objective:**

This study is to evaluate the reliability and validity of SDSC among infants (aged 6–36 months), and to provide a reference for expanding the application of the SDSC for Chinese infants (SDSC-I).

**Materials and methods:**

From April to November 2021, parents of infants from Fuzhou, Quanzhou, Longyan, Sanming, and Nanping cities in Fujian Province, China completed the SDSC-I. Several factor analyses were performed to evaluate the reliability and validity of the scale.

**Results:**

Of note, 432 out of 469 samples were valid. After item selections and exploratory factor analyses, the SDSC-I concluded six dimensions (disorders of initiating sleep, disorders of maintaining sleep, sleep hyperhidrosis, sleep breathing disorders, parasomnias, and non-restorative sleep and excessive somnolence) with 23 items. The Cronbach’s α coefficient of the scale was 0.863, and those for the six dimensions were within 0.576–0.835. The values of parameters for content validity of the scale were: IR = 0.87, I-CVI > 0.78, Kappa value > 0.74, S-CVI/UA = 0.87, S-CVI/Ave = 0.98. Principal component analysis revealed that the Kaiser-Meyer-Olkin (KMO) value was 0.84, and the factor loading of items ranged from 0.328 to 0.849, with six factors of eigenvalue more than one, which could explain 58.274% of the total variance. The confirmatory factor analysis results showed that χ^2^/DF was 3.556, root-mean-square error of approximation (RMSEA) was 0.077, comparative fit index (CFI) was 0.809, and standardized RMR (SRMR) was 0.070.

**Conclusion:**

Our study provides evidence that the SDSC-I is reliable and valid, and it is effective for the screening and management of sleep disturbances among infants (aged 6–36 months). Compared with other questionnaires such as the Brief Infant Sleep Questionnaire (BISQ), it is worthy of popularization and application in pediatric primary care.

## Introduction

Sleep in children is an essential and dynamic process that affects numerous aspects of health and development. It has numerous functions, including growth, development, immune strengthening, memory, learning, regulation of behavior, and emotion. Several important changes in development during the first years of life lead to expected patterns of sleep and wakefulness in adults. Sleep problems are relatively common but they often can be challenging to recognize (1–3). A meta-analysis showed that the prevalence of sleep problems for children in mainland China was 37.6% (95% CI: 34.3–40.9%), with 33.3% in infants, 38.9% in preschoolers, and 43.7% in school-age children (4). Infancy and early childhood are critical periods for the development of sleep and the formation of healthy sleep patterns and habits in children. Few pediatricians in mainland China have received formal training in sleep medicine during medical school or residency. The practitioners have been shown to inadequately address sleep issues during routine well-child healthcare and to underdiagnose sleep problems. As a result, pediatricians are not able to effectively and confidently screen patients for sleep problems and provide sleep-related counseling. Therefore, early screening for sleep problems in infants and toddlers can help to correctly offer the diagnosis and the treatment of sleep disorders.

Since Sen et al. (5) conducted a comprehensive review of the overall deficiencies of pediatric questionnaires in sleep in 2011, many existing tools have been further developed and evaluated by sleep researchers worldwide. There are 144 “tool”-studies (70 tools) published aiming at investigating sleep in primarily 6–18 years old per parental report (5). In 1996, Bruni et al. (6) developed a 26-item Likert-type rating scale (Sleep Disturbance Scale for Children: SDSC) used and validated in an Italian population of 1,304 children. The SDSC appears to be a useful tool for assessing the sleep disturbances of school-age children in both clinical and non-clinical populations. In 2013, they used it to assess the psychometric properties of the SDSC in a population of healthy preschool children (3–6 years old) in Italian. This further demonstrated that the SDSC had good reliability and validity for the application in studies of sleep in young children (7). In the same year, Huang et al. (8) translated the SDSC and determined its reliability and validity in 3,525 children aged 5–16 years. The results showed that its psychometric properties were also satisfactory as original, and indicated that it could be used as an epidemiological screening tool for parent-reported sleep disorders in Chinese school-age children. In 2019, Lecuelle et al. (9) first validated the SDSC for French preschoolers (aged from 6 months to 4 years), and the convergent validity and the internal reliability of a 22-item SDSC for young children (SDSC-Y) were acceptable. Subsequently, Romeo et al. (10) applied a 22-item version of the SDSC to a convenience sample of 193 Italian infants and toddlers (aged 6–36 months), and a 19-item questionnaire was eventually established for the evaluation of sleep problems.

It is believed that the factors affecting childrens sleep are inconsistent, and there is no clear definition of sleep disorders in children. The concepts of sleep disorders and sleep problems are also vague (4, 8). Despite that the studies on children’s sleep in mainland China have gradually increased over the past 20 years, valid assessment tools for pediatric sleep problems are few, and rates of screening and management are low. The SDSC was a parent-report scale for screening sleep disturbances within the preceding 6 months, but it now has been validated and adapted in sleep studies in different languages such as Australian (11), Persian (12), Indonesian (13), Flemish (14), Brazilian Portuguese (15), Portuguese (16), Finnish (17), Malay (18), and Turkish (19). In 2017, the “Guideline for Sleep Hygiene among Children Aged 0–5 Years” was published in mainland China (20), recommending the “Brief Infant Sleep Questionnaire (BISQ)” (21), and the “Children’s Sleep Habits Questionnaire (CSHQ)” (22) as screening tests for children’s sleep. Nevertheless, BISQ was mainly used to assess children’s sleep, such as sleep ecology, infant daytime sleep, nocturnal sleep patterns, bedtime, hours of nighttime, and the number of night wakings (23).

Therefore, this present study was primarily to assess the psychometric properties of the SDSC in a Chinese population of infants and toddlers (6–36 months), which had the potential to assist pediatricians in early screening and diagnosis of sleep disturbances in young children at community hospitals.

## Materials and methods

### Sampling

For from April to November 2021, several community hospitals in Fuzhou, Quanzhou, Longyan, Sanming, and Nanping cities in Fujian Province were selected for sampling using a convenience sampling method. Ethical approval was obtained by the Scientific Research Ethics Committee of Fujian Medical University Union Hospital (approval date and decision number: 2021KY131). And informed consent was acquired from all the parents of the infants who participated in our study.

### Participants

Parents of healthy infants and toddlers aged 6 months to 3 years were selected to exclude children with psycho-behavioral developmental abnormalities, rheumatological and immune diseases, oncological diseases, or the use of medications known to cause sleepiness (e.g., anti-epileptics, antihistamines, and benzodiazepines). Children with developmental, physical, or mental disabilities, or receiving medications such as antihistaminic drugs, antiepileptic drugs, glucocorticoids, and melatonin, were excluded as well, as these factors may affect sleep architecture including sleep cycles of rapid eye movement (REM) and non-rapid eye movement (NREM) sleep. The participants were approached face to face and all the responses were collected digitally.

### The SDSC for the Chinese infants based on the sleep disturbance scale for children

The SDSC adapted in the present study was authorized by Oliviero Bruni. The translation and the adaptation of the scale were performed independently by six senior experts, including a pediatric neurologist, a healthcare physician, a pediatric psychiatrist, a pediatric psychiatrist, an English language specialist, and a nurse. The entries were revised, screened, and evaluated for validation. All specialists independently provided a clarity score between one and four for each item to evaluate the scale’s content validity. Drawing on previous sleep questionnaires, particularly the recent validation of the Italian SDSC (a 19-item version) and the French SDSC-Y (a 22-item version), we finally removed two items (sleepwalking and hypnagogic hallucinations). Meanwhile, the two items “sleep terrors” and “nightmares” were combined into one (sleep terrors or nightmares). And an item (Need of parental accompaniment for sleep) was added, resulting in a 23-item questionnaire. This questionnaire investigates the occurrence of sleep disturbance during the preceding 6 months and each item is scored on a 5-point Likert scale. A sociodemographic data form was filled out by the parents, which included the date of birth, gender, developmental history, and medical history (i.e., past or present diseases, therapies, etc.) of the children. All the respondents were the mothers of the children.

### Statistical analysis

The normality of the data was examined by using the Shapiro–Wilks test. Arithmetic means ± standard deviations of item scores were reported as the previous study reported (10). Item analysis was used to evaluate the appropriateness of the items. The subjects were ranked in descending order of their total scores. The content validity of the scale was evaluated using inter-rater reliability (IR), item-content validity index (I-CVI), scale-content validity index (S-CVI), universal agreement S-CVI (S-CVI/UA), average S-CVI (S-CVI/Ave), and Kappa value (24–26). The reliability analysis of the scale was evaluated by Cronbach’s alpha, inter-item correlation, and internal correlation consistency. The T-score table was prepared similarly to that in the original study by converting the mean scores of the scale into T-scores using the following formula: T-score = 50 + (value-mean)/standard deviation*10. A child with a T-score of more than 70 was considered to have a significant sleep disorder (14). Confirmatory factorial analyses (CFA) were performed by a structural equation model. Comparative fit index (CFI) value >0.80 was considered a moderate fit (27). The values of root-mean-square error of approximation (RMSEA) and standardized RMR (SRMR) < 0.08 were accept (28). The data analysis was performed using the Statistical Product and Service Solutions (SPSS) version 21.0 and Mplus version 8.3 software, and *p* < 0.05 was considered to be statistically significant.

## Results

### Participants

The ages of the 432 children ranging from 6 months to 3 years, and the average was 2 years and 1 month ± 7 months. The mean age for 242 boys (56.02% of total) was 2 years with a standard deviation of 6 months, and the mean age for 190 girls (43.98% of total) was 2 years with a standard deviation of 7 months. A total of 188 of the participants (43.5%) were infants younger than 24 months. They were from a non-deprived population relative to the norm because the average household income of our population was more than 4,000 yuan/year. Other characteristics of the sample are listed in Table 1.

**TABLE 1:** Characteristics of the sample (*n* = 432).

Characteristic		*n*	%
Region	Rural	226	52.30
	Urban	206	47.70
Household income	<10,000 yuan	37	8.60
	10,000–5,000 yuan	120	27.80
	50,000–100,000 yuan	160	37.00
	>100,000 yuan	115	26.60
Mother’s education	Primary school and lower	90	20.80
	Junior high school	92	21.30
	High school or junior college	246	56.90
	College or university	4	0.90
Mother’s work	Unemployed	125	28.90
	Full-time	270	62.50
	Part-time	37	8.60
Housing area per capita	<10 m^2^	34	7.90
	10–30 m^2^	231	53.50
	>30 m^2^	167	38.70

### Item analysis

Based on the total scores, the critical ratio method was used to divide 27% into the “high” group (*n* = 132, ≥48 points, numbered 2) and the “low” group (*n* = 129, ≤37 points, numbered 1), and *t*-tests were conducted to determine the differences between the two groups on each entry score. The results showed statistically significant differences between the high and the low subgroups (*p* < 0.05, see Table 2), indicating a strong differentiation in each dimension.

**TABLE 2:** Item analysis for the Chinese version of SDSC-I (*n* = 432).

Item	High group	Low group	*T*	*P*
D1	1.61 ± 0.63	1.27 ± 0.48	4.85	<0.001
D2	3.30 ± 1.09	1.95 ± 0.77	11.56	<0.001
D3	2.99 ± 1.17	1.47 ± 0.64	13.14	<0.001
D4	3.12 ± 1.15	1.35 ± 0.53	16.04	<0.001
D5	4.76 ± 0.59	3.28 ± 1.58	9.98	<0.001
D6	4.36 ± 1.12	2.10 ± 1.47	13.93	<0.001
M7	2.80 ± 1.30	1.45 ± 0.64	10.73	<0.001
M8	2.05 ± 1.04	1.09 ± 0.28	10.35	<0.001
M9	4.26 ± 0.88	2.26 ± 1.24	14.95	<0.001
H10	3.03 ± 1.28	1.29 ± 0.58	14.27	<0.001
H11	2.67 ± 1.28	1.19 ± 0.45	12.56	<0.001
B12	1.39 ± 0.75	1.00 ± 0.00	6.04	<0.001
B13	1.15 ± 0.50	1.00 ± 0.00	3.47	0.001
B14	1.97 ± 1.02	1.20 ± 0.42	7.99	<0.001
P15	1.99 ± 1.01	1.21 ± 0.41	8.26	<0.001
P16	2.38 ± 0.94	1.30 ± 0.49	11.65	<0.001
P17	1.86 ± 0.91	1.23 ± 0.46	7.02	<0.001
P18	1.48 ± 0.78	1.10 ± 0.33	5.23	<0.001
N19	1.85 ± 1.13	1.16 ± 0.56	6.25	<0.001
N20	1.70 ± 0.86	1.04 ± 0.23	8.56	<0.001
N21	1.52 ± 0.84	1.02 ± 0.15	6.60	<0.001
N22	1.68 ± 0.86	1.09 ± 0.32	7.38	<0.001
N23	1.55 ± 0.78	1.21 ± 0.51	4.24	<0.001

### Content validity

The results showed as follows: the inter-rater reliability of the SDSC-I = 0.87, I-CVI > 0.78, Kappa values > 0.74, S-CVI/UA = 0.87, and S-CVI/Ave = 0.98. All values met the requirements. The content validity of the scale was desirable, presented in Table 3.

**TABLE 3:** Content validity analysis of each item for the Chinese version of SDSC-I (*n* = 432).

Item	Expert rating (score)						I -CVI	Kappa
			
	A	B	C	D	E	F		
D1	4	3	4	4	4	4	1.00	1.00
D2	4	4	4	4	4	4	1.00	1.00
D3	3	3	4	3	3	4	1.00	1.00
D4	4	4	3	3	3	4	1.00	1.00
D5	3	3	4	2	4	4	0.83	0.81
D6	3	2	4	3	4	3	0.83	0.81
M7	4	3	4	3	4	3	1.00	1.00
M8	4	4	4	3	4	3	1.00	1.00
M9	4	4	4	4	4	3	1.00	1.00
H10	4	4	4	4	3	3	1.00	1.00
H11	4	4	4	3	3	4	1.00	1.00
B12	3	3	4	4	3	3	1.00	1.00
B13	3	3	4	4	4	3	1.00	1.00
B14	4	4	4	3	4	3	1.00	1.00
P15	4	4	4	2	4	4	0.83	0.81
P16	4	4	4	3	4	4	1.00	1.00
P17	4	4	4	3	4	4	1.00	1.00
P18	4	3	4	4	3	4	1.00	1.00
N19	4	4	4	3	4	4	1.00	1.00
N20	4	4	4	3	4	4	1.00	1.00
N21	3	3	4	3	4	3	1.00	1.00
N22	4	4	4	3	3	4	1.00	1.00
N23	4	4	4	3	4	4	1.00	1.00

### Reliability analysis

The Cronbach’s alpha value for the scores of 23 items was 0.838, indicating good reliability of the scale. The Cronbach’s alpha value for all dimensions ranged from 0.576 to 0.835. Except for Item 1 and Item 24, item-total correlations were greater than 0.3. After eliminating Item 1 or Item 24, the alpha was unchanged (see Table 4).

**TABLE 4:** Item-total correlation analysis and exploratory factor analysis of the SDSC-I (*n* = 432).

	Item-total correlation	Alpha if deleted	Variance explained (%)	Factor loading
Factor 1: Disorders of initiating sleep (DIS)			11.747	
D1. Sleep duration	0.155	0.839		0.463
D2. Sleep latency	0.423	0.830		0.328
D3. Going to bed reluctantly	0.511	0.826		0.679
D4. Difficulty in falling asleep	0.610	0.821		0.721
D5. Need of parental accompaniment for sleep	0.351	0.835		0.849
D6. Anxiety when falling asleep alone	0.412	0.835		0.817
Factor 2: Disorders of Maintaining Sleep (DMS)			11.129	
M7. Night awakenings more than twice	0.431	0.830		0.586
M8. Difficulty in falling asleep after waking	0.518	0.827		0.660
M9. Nocturnal hyperkinesia	0.473	0.829		0.419
Factor 3: Sleep Hyperhidrosis (SHY)			10.994	
H10. Falling asleep sweating	0.473	0.828		0.837
H11. Night sweating	0.497	0.827		0.824
Factor 4: Sleep Breathing Disorders (SBD)			9.061	
B12. Breathing problems	0.344	0.834		0.765
B13. Sleep apnea	0.229	0.837		0.806
B14. Snoring	0.418	0.831		0.603
Factor 5: Parasomnias (PARA)			8.112	
P15. Startles or jerks while falling asleep	0.456	0.830		0.668
P16. Sleep terrors or nightmares	0.551	0.826		0.735
P17. Sleep talking	0.368	0.833		0.545
P18. Sleep bruxism	0.257	0.836		0.421
Factor 6: Non-restorative sleep and excessive somnolence (NRSES)			7.232	
N19. Unusually difficult to wake up in the morning	0.282	0.836		0.638
N20. Feeling tired with non-restorative sleep	0.445	0.831		0.787
N21. Sleep paralysis	0.387	0.833		0.773
N22. Daytime somnolence	0.381	0.833		0.664
N23. Sleep attacks	0.276	0.845		0.543

A Shapiro–Wilks test of the total scale and subscale scores demonstrated a non-normal distribution. Non-parametric Spearman correlations confirmed these results. All correlations between the two subscales (DIS, DMS, SHY, SBD, PARA, and NRSES) of the SDSC-I were smaller than 0.40, except for the correlation between DIS and DMS (0.525). Between the total scores and the subscales, a strong correlation was observed ranging from 0.501 (SBD) to 0.820 (DIS).

### Construct validity

**FIGURE 1:**
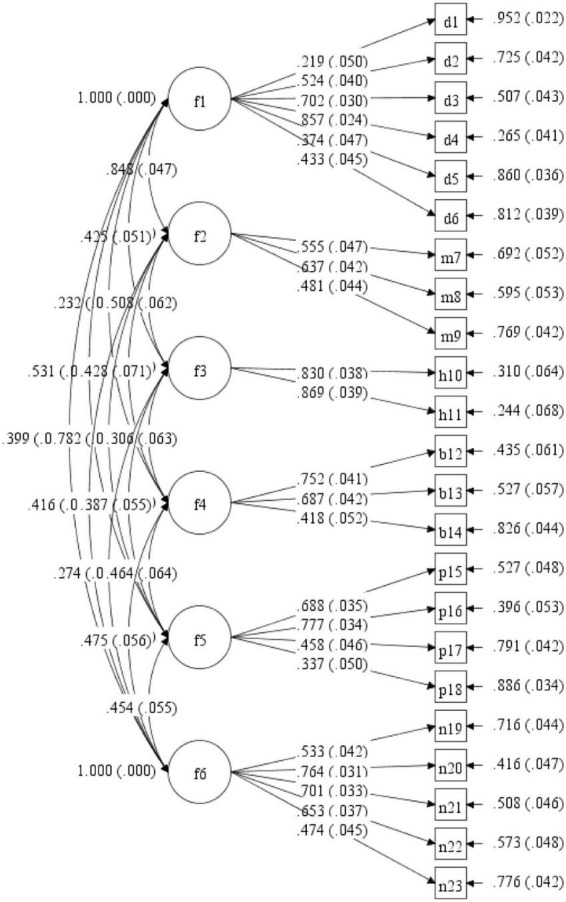
Model path analysis diagram.

This scale was suitable for exploratory factor analysis (EFA) using geomin rotation, with a Kaiser-Meyer-Olkin (KMO) value of 0.82 and Bartlett’s test of sphericity of χ^2^ value of 3,088.36 (*p* < 0.001). Based on the eigenvalues, a six-factor solution explained 58.27% of the total variance. The factor loads of all the items varied between 0.328 (Item 2) and 0.849 (Item 5). The 23-item scale, the factor loadings, and the six factors were depicted in Figure 1. In the model described in CFA, values were determined as follows: χ^2^/DF = 3.556, CFI = 0.809, SRMR = 0.070, and the RMESA = 0.077. The factor loadings of the 23 items were from 0.22 to 0.87 (see Figure 1). The factor structure of the Chinese SDSC in infants was consistent with previous studies and appeared to be compatible.

### Scale score distribution

**FIGURE 2:**
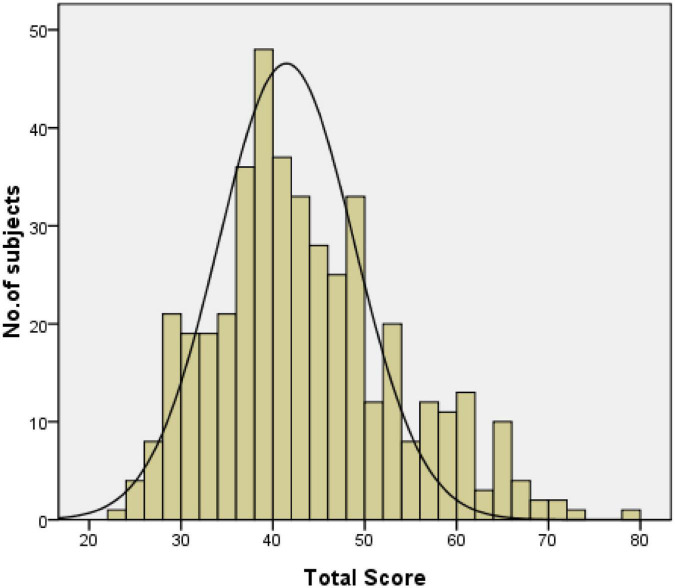
Distribution of the SDSC-I total score.

The minimum score of the scale was 23, and the minimum scores for the subscales of DIS, DMS, SHY, SBD, PARA, and NRSES were 6, 3, 2, 3, 4, and 5, respectively. The distribution of SDSC scores showed the maximum scores of the total scale and subscales were 78, 27, 15, 10, 11, 15, and 20, respectively. The single factor scores showed the percentages of the sample with minimum and maximum values ranging from 0.20 to 0.20% for DIS, 0.70 to 0.70% for DMS, 6.5 to 0.20% for SHY, 37.5 to 3.5% for SBD, 58.3 to 0.20% for PARA, 22.2 to 0.70% for NRSES, and 48.8 to 0.20% for T-score. All the total scores demonstrated left-tailed distribution (Wilk’s *W* = 0.973, *p* = 0.000) (see Figure 2). Differences in total scores by age and gender were not found.

### Other measurements

As previously done in similar studies (10), most of the infants (62.0%) slept more than 9 h per night and none slept less than 5 h. A total of 59.7% of the young children fell asleep 30 min after going to bed. The highest frequency of parent-reported sleeping disturbance was Item 5 (77.3%), and the lowest ones were Item 1 (0.2%) and Item 13 (0.2%) (see Table 5). The analysis of the participant’s compliance and the completion time of the scale showed that most of the parents chose to fill out the questionnaire issued in this study and completed it within 10–15 min, and the recovery rate of the questionnaire was over 90%.

**TABLE 5:** Frequencies (%) of parent-reported sleeping disturbances in infants (*n* = 432).

Item	Never	Occasionally 1–2x/month	Sometimes 1–2x/week	Often 3–5x/week	Always
D3	32.200	37.300	16.900	8.600	5.100
D4	33.600	37.300	15.300	9.300	4.600
D5	6.900	8.800	6.900	14.800	62.500
D6	23.400	14.800	8.300	12.700	40.700
M7	37.300	38.900	9.700	9.700	4.400
M8	64.100	25.000	7.900	2.300	0.700
M9	11.100	22.200	12.300	30.600	23.800
H10	42.400	26.900	13.900	9.000	7.900
H11	50.200	27.800	10.600	7.400	3.900
B12	88.200	7.600	2.800	0.900	0.500
B13	95.600	3.000	1.200	0.200	0.000
B14	62.000	28.000	6.700	2.300	0.900
P15	55.800	35.600	4.900	3.000	0.700
P16	40.500	44.400	10.600	4.200	0.200
P17	58.100	32.900	6.900	2.100	0.000
P18	78.900	16.400	3.500	1.200	0.000
N19	73.600	14.400	7.200	3.000	1.900
N20	78.200	15.700	4.600	1.400	0.000
N21	85.200	10.900	3.000	0.500	0.500
N22	75.900	18.800	4.200	0.900	0.200
N23	74.100	19.200	5.600	1.200	0.000

## Discussion

The main objective of the present study was to assess sleep disturbance in Chinese infants and toddlers using the SDSC. The validity and reliability were investigated by a modified version of SDSC-I. The 23-item SDSC-I consisted of six factors (DIS, DMS, SHY, SBD, PARA, and NRSES) and was highly reliable and compatible.

Our study demonstrated that there was a strong correlation between each dimension of the SDSC-I scale and the total score, with all the correlations more than 0.5. It indicated that the factors (DIS, DMS, SHY, SBD, PARA, and NRSES) were consistent with the clinical concept, as previously reported (9, 10). Except for Item 1, the correlation coefficient values were all greater than 0.5 between each item and the corresponding dimension. Therefore, our study showed that the dimensions in which the entries were located were reasonable and suggested that the scale had good construct validity. According to the exploratory factor analyses, six factors with eigenvalues greater than one were extracted, which was basically consistent with the findings from the Italian and French scholars (9, 10). And all six areas of sleep disorders based on clinical concepts and previous studies were helpful for clinicians to identify the areas that require more in-depth investigations (10). All of the factor loadings of items ranged from 0.328 to 0.849, which was similar to the results (from 0.26 to 0.91) in the previous French study and slightly lower than the results (from 0.50 to 0.87) in the Italian study. Six-factor model in the present study matched the theoretical constructs of the six dimensions designed by the SDSC-I, indicating that the scale had good structural validity.

In the present study, it was the first time that the SDSC was applied to infants and toddlers within 3 years old in mainland China. Sleep problems are prevalent in Chinese preschool and school-age children, including difficulties in falling asleep, enuresis, night waking, teeth grinding, sleep breathing disorder, night terrors, open mouth breathing, sleep talking, nightmares, night sweats, and daytime sleepiness (29). The existing sleep assessment tools for children are mainly applicable to preschool or school-age children, and most of them are limited to the assessment of one specific sleep problem but cannot accurately and comprehensively reflect the sleep problems of infants in China (30). The clinical evidence demonstrated that pediatric sleep disturbances overlapped with each other (10). The SDSC not only evaluates sleep duration and sleep behavior habits but also assesses sleep disorder-related problems, which is more beneficial for the early screening of sleep disorders in clinical practice. The SDSC is currently used in China to only evaluate school-age children aged from 5 to 16 years (8), but it has been used to assess preschool-age children or infants in other countries (9, 10). The validity and reliability of the Chinese version of SDSC for children in mainland China first conducted by Huang et al. (8) revealed a good internal consistency (Cronbach’s α = 0.81). Our study showed that the SDSC-I scale had an acceptable and stable internal consistency with a Cronbach’s α coefficient of 0.847, slightly higher than the result of previous research from Chinese scholars (8), but it was similar to the French population and the Italian population (9, 10). The internal consistency of all the dimensions was observed across infants and school-age children in China (8). Therefore, this study indicated that the SDSC-I had good reliability for evaluating sleep disturbance in Chinese infants.

The current international classification of sleep disorders is based on the “The Third Edition of the International Classification of Sleep Disorders (ICSD-3)” released in 2014, identifying seven categories (31) including insomnia disorders, sleep-related breathing disorders, central disorders of hypersomnolence, circadian rhythm sleep–wake disorders, parasomnias, sleep-related movement disorders, and other sleep disorders. Insomnia in children is usually reported by parents or other caregivers and is mainly characterized by going to bed reluctantly, frequent awakenings during the night, and difficulty falling asleep alone. In addition to nocturnal symptoms, daytime symptoms of functional impairment such as fatigue, irritability, hyperactivity and impulsivity, and daytime sleepiness may also result from night sleep difficulties (32). The 23-item version of the SDSC in our study was also an adapted version of the original SDSC as Lecuelle et al. (9) and Romeo et al. reported (10). In the SDSC-I, we removed three items regarding sleepwalking and hypnagogic hallucinations. Bruxism, on the other hand, was retained in our study due to the results of research on sleep problems in children in mainland China (4, 29, 30). Because, even though bruxism is reported to be poorly prevalent in French and Italian infants and toddlers, it is common among infants and young children in mainland China (29). Furthermore, in French, rhythmic movement disorders and hypnic jerks are frequently misinterpreted by parents as that the child is agitated or turning several times in bed when unable to fall asleep (9), but parents, as well as researchers of sleep problems in mainland China, are more concerned about children’s frequency of awakenings, restless sleep, leg movements, bruxism, hyperhidrosis, snoring, night awakening, apnea, choking or gasping, mouth breathing, sleep talking, nightmare, enuresis, trouble falling asleep, or other problems during sleep (4). For instance, Chinese parents of young children pay particularly close attention to bruxism as they often associate bruxism with ascariasis or other diseases. Therefore, bruxism was not removed after consultation with specialists and parents. Despite that the national and cultural differences led to slight inconsistency in the application of the SDSC, the scale still illustrated good applicability.

Compared with the previous study by Romeo et al. (10), 58.5% of infants in our group slept 9–11 h, which was higher than the Italian population (46.6%). A sleep latency shorter than 30 min to fall asleep was about 59.7% of the Chinese infants, rather than 54.5% of the Italian infants. The incidence of bedtime resistance and difficulty in falling asleep among Chinese infants were lower than those in Italian infants (13.6 vs. 20.9% and 13.2 vs. 16.6%, respectively). Chinese infants and toddlers were awakened less frequently during the night (13.6 vs. 20.9%) and presented nocturnal hyperkinesia (13.2 vs. 16.6%). Snoring was found similar in the Chinese and Italian populations (5.7 vs. 4.8%). As for parasomnias, a prevalence of 3.1% of sleep talking was reported in our research while 3.5% in the Italian research and frequent twitching or jerking of legs were reported in 54.4% of Chinese infants, significantly higher than in Italian (24.0%).

The practice of parent and child sharing a sleeping surface, or “bed-sharing,” is one of the most controversial topics in parenting research. The lay literature has popularized and polarized this debate, offering claims of dangers and benefits, both physical and psychological, associated with bed-sharing (33). The bed-sharing practice is very prevalent at any age, which ranges from 69.9 to 78.3%. Most infants fall asleep while feeding or being rocked/held before age 12 months. By the age of 35 months, 62.4% of the children fall asleep in bed near their parents. The most common reasons for bed-sharing are breastfeeding/feeding and convenience. Parental involvement when falling asleep is significantly related to frequent night awakenings and difficulty falling asleep. Bed-sharing and parental involvement were very common among Chinese children aged <years (34). This may be due to the fact that Chinese parents prefer to share a bed with their children for sleep, as studies have shown that Chinese children usually need to be held or patted to sleep by their parents (35). The percentages of sweating while falling asleep and during sleep were respectively 16.9% (vs. 7.7% in Italian) and 11.3% (vs. 8.4% in Italian). It may be related to the high parental concern about night sweating or excessive warmth of children in mainland China. Interestingly, parents of Chinese infants often associate excessive sweating with vitamin D deficiency or physical weakness (a symptom defined in traditional Chinese medicine). The percentage of T-score greater than 70 was higher than that in the Italian population (4.6 vs. 3.6%) and similar to that of Chinese school-age children in the previous study (4.1%). Our study showed that 10.9% of infants and toddlers were within the critical range of 61–70 points, while 12% in the Italian study were within that critical value. The influencing factors relevant to sleep disorders or sleep problems in children vary widely from study to study and may be closely related to sociocultural, climatic environment, and individual or family factors (29).

Several limitations of our study need to be acknowledged as follows: (a) the sensitivity and specificity of the scale could not be calculated in our study because of lacking a patient group with sleep disorders. Due to methodological issues, the rates of sleep disorders determined in this study cannot be used as epidemiological data (19); (b) the parents may lack awareness and attention to sleep problems because of social, familial, cultural, and environmental factors. Therefore, data collected from parental reports had a potential recall bias (8); and (c) our study also has some statistical validation limitations such as lacking test–retest fidelity, comparisons of different age groups, and multi-group modeling CFA (9). Nevertheless, the potential sleep-related problems can be evaluated by pediatricians by easily administrating, scoring, and interpreting the SDSC-I, which would be conducive to addressing the prevention of sleep disorders (8).

## Conclusion

Our study provides evidence that SDSC-I is a reliable and valid version as qualified as in other languages, and it can assess the sleep problems of infants in a comprehensive and detailed manner. Therefore, this scale is useful for screening and detection of sleep disturbances in pediatric primary care and is worthy of popularization and application. This study is the first step to applying the SDSC to Chinese infants, and further research is needed to provide a more in-depth assessment of sleep disorders like polysomnography or actigraphy. Recommendations for improving sleep services in pediatric primary care are also needed in the areas of research, practice, and education.

## Data availability statement

The original contributions presented in this study are included in the article/supplementary material, further inquiries can be directed to the corresponding authors.

## Ethics statement

The studies involving human participants were reviewed and approved by the Fujian Medical University Union Hospital, Scientific Research Ethics Committee (approval date and decision number: 2021KY131). Written informed consent to participate in this study was provided by the participants’ legal guardian/next of kin.

## Author contributions

XC and XL participated in the design of the study and data collection and performed the statistical analysis. XL, XC, YC, YY, PX, and SC conceived of the study and participated in its design as well as coordination and helped to write and revise the manuscript. All authors read and approved the final version of the manuscript.

## Abbreviations

SDSC: sleep disturbance scale for children
SDSC-I: SDSC for the Chinese infants
EFA: exploratory factor analysis
CFA: confirmatory factorial analyses
SDSC-Y: a French version of the SDSC adapted for preschool (6-month to 4-year-old) children
BISQ: Brief Infant Sleep Questionnaire
CSHQ: Children’s Sleep Habits Questionnaire
IR: inter-rater reliability
I-CVI: item-content validity index
S-CVI: scale lever-content validity index
CFI: comparative fit index
RMSEA: root-mean-square error of approximation
SRMR: standardized RMR
DIS: disorders of initiating sleep
DMS: disorders of maintaining sleep
SHY: sleep hyperhidrosis
SBD: sleep breathing disorders
PARA: parasomnias
NRSES: non-restorative sleep and excessive somnolence
ICSD-3: the third edition of the international classification of sleep disorders
KMO: Kaiser-Meyer-Olkin
SPSS: Statistical Product and Service Solutions.
